# *QuickStats:* Percentage of Adults Aged ≥20 Years Who Used Antidepressant Medications* in the Past 30 Days, by Sex and Marital Status — National Health and Nutrition Examination Survey, United States, 2015–2018

**DOI:** 10.15585/mmwr.mm6942a8

**Published:** 2020-10-23

**Figure:**
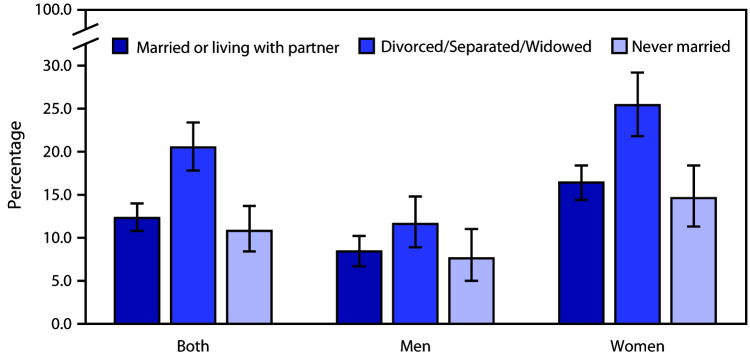
During 2015–2018, 13.6% of adults aged ≥20 years used prescription antidepressant medications in the past 30 days. Antidepressant use was higher among divorced, separated, or widowed (20.5%) adults than among either married or living with partner (12.3%) or never married (10.8%) adults. There was no difference in use between married and never married adults. These same patterns were observed for both men and women. Within every marital status category, a higher percentage of women compared with men took antidepressants.

**Source:** Brody DJ, Gu Q. Antidepressant use among adults: United States, 2015–2018. NCHS Data Brief, no 377. Hyattsville, MD: National Center for Health Statistics. 2020.

